# HMGB1 assists the predictive value of tumor PD-L1 expression for the efficacy of anti-PD-1/PD-L1 antibody in NSCLC

**DOI:** 10.1007/s00280-025-04751-2

**Published:** 2025-01-24

**Authors:** Kunihiko Funaishi, Kakuhiro Yamaguchi, Hiroki Tanahashi, Koji Kurose, Shinjiro Sakamoto, Yasushi Horimasu, Takeshi Masuda, Taku Nakashima, Hiroshi Iwamoto, Hironobu Hamada, Toru Oga, Mikio Oka, Noboru Hattori

**Affiliations:** 1 Department of Molecular and Internal Medicine, Graduate School of Biomedical and Health Sciences, Hiroshima University, Hiroshima, Japan; 2 Department of Respiratory Medicine, Hiroshima University Hospital, 1-2-3 Kasumi, Minami-ku, Hiroshima, 734-8551 Japan; 3 Department of Respiratory Medicine, Kawasaki Medical School, Okayama, Japan; 4 Department of Physical Analysis and Therapeutic Sciences, Graduate School of Biomedical and Health Sciences, Hiroshima University, Hiroshima, Japan; 5 Department of Immuno-Oncology, Kawasaki Medical School, Okayama, Japan

**Keywords:** Immune checkpoint inhibitor, Biomarker, HMGB1, Prediction, PD-L1

## Abstract

**Background:**

The expression of anti-programmed cell death ligand-1 (PD-L1) in tumors is widely used as a biomarker to predict the therapeutic efficacy of anti-programmed cell death-1(PD-1)/PD-L1 antibodies. However, the predictive accuracy of this method is limited. High-mobility group box 1 (HMGB1) is known to modulate cancer immunity. Therefore, we investigated the potential of circulatory HMGB1 in combination with PD-L1 expression to predict the efficacy of anti-PD-1/PD-L1 antibody monotherapy.

**Patients and methods:**

This multicenter retrospective study analyzed blood samples collected from 114 patients with non-small cell lung cancer (NSCLC) prior to anti-PD-1/PD-L1 antibody monotherapy at two university hospitals (Hiroshima University Hospital and Kawasaki Medical School Hospital) between December 2015 and October 2020. We evaluated the association of serum HMGB1 levels with tumor response and progression-free survival (PFS).

**Results:**

Serum HMGB1 levels were significantly higher in patients with complete or partial response than in those with stable or progressive disease. Using receiver operating characteristic analysis, the cut-off level of serum HMGB1 to predict tumor response was determined to be 3.83 ng/mL. PFS was significantly longer in the HMGB1^high^ group than that in the HMGB1^low^ group in the entire cohort (4.3 months vs. 2.3 months) and in patients with NSCLC with PD-L1 tumor proportion score (TPS) ≥ 50% (12.4 months vs. 4.4 months), but not in those with PD-L1 TPS < 50% or unknown.

**Conclusion:**

HMGB1 may serve as a predictive biomarker for the efficacy of anti-PD-1/PD-L1 antibody therapy in the patients with NSCLC, especially in those with PD-L1 TPS ≥ 50%.

**Supplementary Information:**

The online version contains supplementary material available at 10.1007/s00280-025-04751-2.

## Introduction

Immune checkpoint inhibitors (ICI), such as anti-programmed cell death-1 (PD-1) and anti-programmed cell death ligand-1 (PD-L1) antibodies, have improved the prognosis of patients with non-small cell lung cancer (NSCLC) [1]. PD-L1 expression in tumors is widely used as a predictive biomarker for therapeutic efficacy of anti-PD-1/PD-L1 antibodies. The tumor proportion score (TPS) of PD-L1 evaluated using the anti-PD-L1 antibody 22C3 stratified progression-free survival (PFS) and objective response rate (ORR) in patients with NSCLC treated with pembrolizumab [2]. However, the predictive accuracy is insufficient. The KEYNOTE-024 trial revealed that the ORR was 44.8% in patients with NSCLC with PD-L1 TPS ≥ 50% [1]. Therefore, additional predictive biomarkers that may be used along with PD-L1 TPS in selecting patients who are likely to benefit from ICI therapy need to be identified.

High-mobility group box 1 (HMGB1) is a nuclear protein that is involved in DNA organization and transcriptional regulation [3]. HMGB1 is detectable in circulation as it is released passively during cellular necrosis by almost all cells and is actively secreted by immune cells such as monocytes, macrophages, and dendritic cells (DC) [4]. Once released extracellularly, HMGB1 acts as an alarmin and stimulates natural host defense [5–7]. HMGB1 is also known to modulate cancer immunity, although conflicting data exist; HMGB1 plays an immunosuppressive role by facilitating the differentiation of myeloid-derived suppressor cells, activating regulatory T cells, and recruiting tumor-associated macrophages [8]. HMGB1 also reportedly enhances anti-tumor immune response by stimulating mature DC tumor antigen procesing [9]. Therefore, we hypothesized that HMGB1 may be a predictive biomarker for response to anti-PD-1/PD-L1 antibody therapy; however, no study has yet elucidated their association.

This study aimed to investigate whether serum HMGB1 levels prior to the first administration of an anti-PD-1/PD-L1 antibody may serve as a predictive blood marker for the efficacy of anti-PD-1/PD-L1 antibody monotherapy in patients with NSCLC.

## Methods

### Study population and design

This multicenter study consecutively enrolled patients with advanced NSCLC who were treated with anti-PD-1/PD-L1 antibody monotherapy (nivolumab, pembrolizumab, or atezolizumab) between December 2015 and October 2020 at Hiroshima University Hospital and Kawasaki Medical School Hospital. We excluded patients whose blood samples were not stored. This study also excluded the patients with preexisting interstitial lung disease (ILD) or whose treatment efficacy could not be evaluated using the Response Evaluation Criteria in Solid Tumors (RECIST).

This study was approved by the Ethics Committee of Hiroshima University Hospital and Kawasaki Medical School Hospital. All participants provided written informed consent for the collection of blood samples and the use of medical records (Hiroshima University Hospital [M326-19 and E-2146], and Kawasaki Medical School Hospital [#2071-10]).

### Evaluation of anti-tumor effects and PFS

The anti-tumor effect of the anti-PD-1/PD-L1 antibody was assessed using RECIST version 1.1. PFS was defined as the time from the first administration of the anti-PD-1/PD-L1 antibody to disease progression or death.

### Measurement of serum HMGB1 concentration

Serum samples were collected prior to the first administration of anti-PD-1/PD-L1 antibody at Hiroshima University Hospital and Kawasaki Medical School Hospital and stored at -80 °C. Serum HMGB1 levels were measured using commercially available enzyme-linked immunosorbent assay kits (Shino-Test Corporation, Tokyo, Japan), according to the manufacturer’s instructions.

### Statistical analyses

Values are expressed as median (interquartile range). Differences among groups were examined using the Mann-Whitney *U* test and Pearson’s chi-squared test. Cox proportional hazards analysis was used to identify significant predictors of the 2-year PFS. Receiver operating characteristic (ROC) curve analysis was performed to determine the optimal cut-off level of serum HMGB1. PFS was evaluated using the Kaplan-Meier approach and the log-rank test. Censored cases were defined as patients who were lost to follow-up. Statistical significance was set at *p* < 0.05. All data analyses were performed using the JMP statistical software (version 16.0.0, SAS Institute Inc., Cary, NC, USA).

## Results

### Patient enrollment

**Fig. 1:**
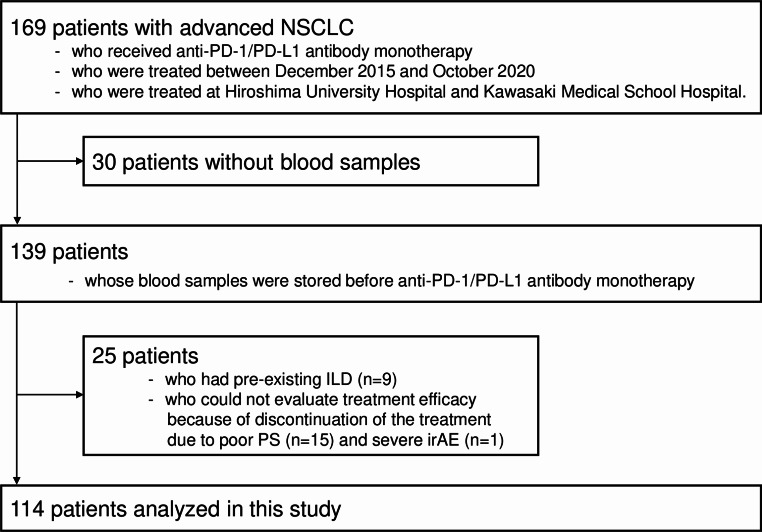
Flowchart of patient enrollment. This study consecutively included 169 advanced NSCLC patients who were treated with anti-PD-1/PD-L1 antibody monotherapy between December 2015 and October 2020 at two University Hospital. Among these, blood samples were prospectively stored before treatment from 139 patients. After excluding 25 patients who had pre-existing ILD or who could not evaluate treatment efficacy, 114 patients were analyzed in this biomarker study

As shown in Fig. 1, this study consecutively enrolled 169 patients. Blood samples were collected and stored from 139 patients before treatment. Twenty-five patients with preexisting ILD or whose treatment efficacy could not be evaluated were excluded. Ultimately, 114 patients were included in this retrospective biomarker study.

### Patient characteristics

The baseline characteristics of the study population are shown in Table 1. The majority of patients were male and had a history of smoking. Of the 114 patients, 84 (74%) were histologically diagnosed with non-squamous NSCLC and 88 (77%) were treated with anti-PD-1/PD-L1 antibody monotherapy in second- or later-line settings. The percentage of patients with NSCLC with PD-L1 TPS ≥ 50% was 32% (36 out of 114 patients).

**Table 1:** Baseline characteristics (*n* = 114)

Age, years	69 (64–76)
Sex	
Male, n (%)	85 (75)
Female, n (%)	29 (25)
Smoking history	
Never, n (%)	20 (18)
Current or Former, n (%)	94 (82)
PS	
0–1, n (%)	92 (81)
≥ 2, n (%)	22 (19)
Stage	
III, n (%)	14 (12)
IV, n (%)	71 (62)
Recurrence, n (%)	29 (26)
Histological type	
Sq, n (%)	30 (26)
Non-Sq, n (%)	84 (74)
PD-L1 TPS	
≥ 50%, n (%)	36 (32)
< 50% or Unknown, n (%)	78 (68)
Line	
1st, n (%)	26 (23)
2nd or later, n (%)	88 (77)
ICI agent	
Nivo, n (%)	52 (46)
Pembro, n (%)	52 (46)
Atezo, n (%)	10 (8)

Data are presented as median (interquartile range) unless stated otherwise

Atezo, atezolizumab; ICI, immune checkpoint inhibitor; Sq, squamous cell carcinoma; Nivo, nivolumab; PD-L1, programmed death ligand 1; Pembro, pembrolizumab; PS, performance status; TPS, tumor proportion score

### Association between serum HMGB1 levels and ORR

**Fig. 2:**
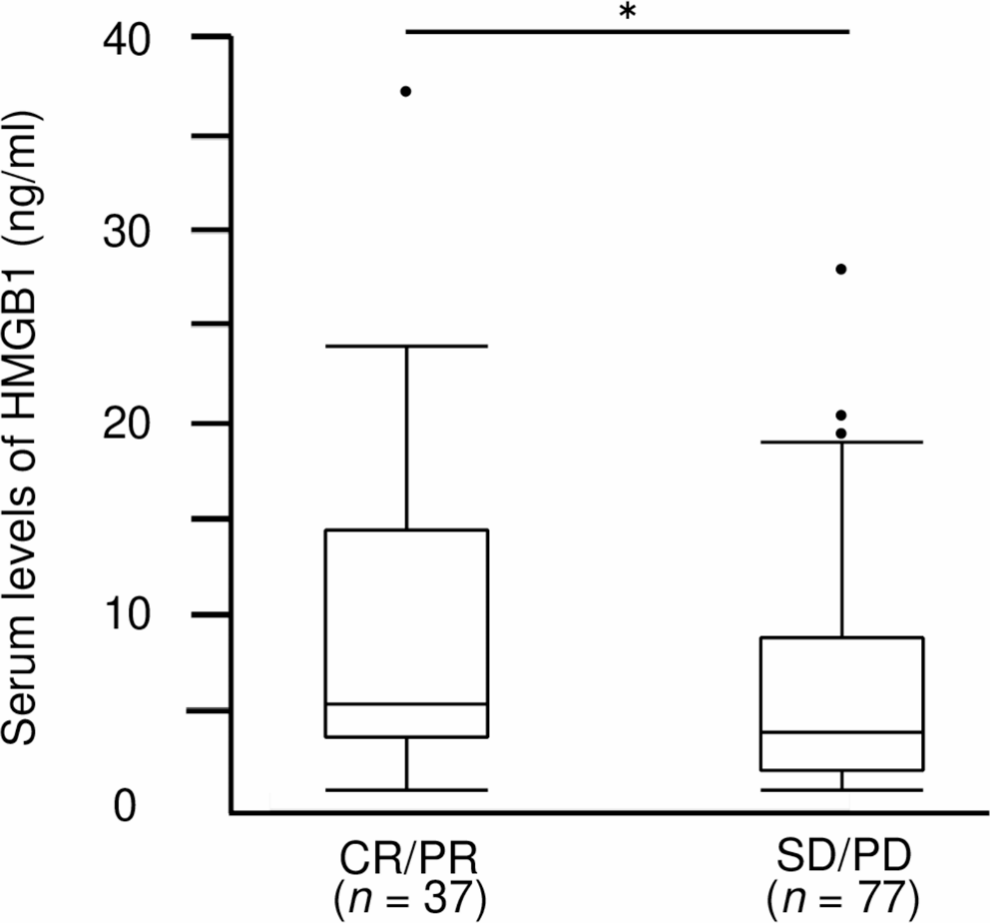
Serum high-mobility group box 1 (HMGB1) levels based on the tumor response to anti-PD-1/PD-L1 antibody. Serum HMGB1 levels in patients with complete response (CR) or partial response (PR) were significantly higher than those in patients with stable disease (SD) or progressive disease (PD) (5.26 ng/ml [3.62–13.94] vs. 3.87 ng/ml [1.99–8.52], *p* = 0.02). Boxes represent the 25th to 75th percentiles; solid lines within the boxes show the median values; whiskers represent the 10th and 90th percentiles; the dots represent outliers. * *p* < 0.05 using the Mann-Whitney *U* test. CR, complete response; PR, partial response; SD, stable disease; PD, progressive disease; HMGB1, high-mobility group box 1

**Fig. 3:**
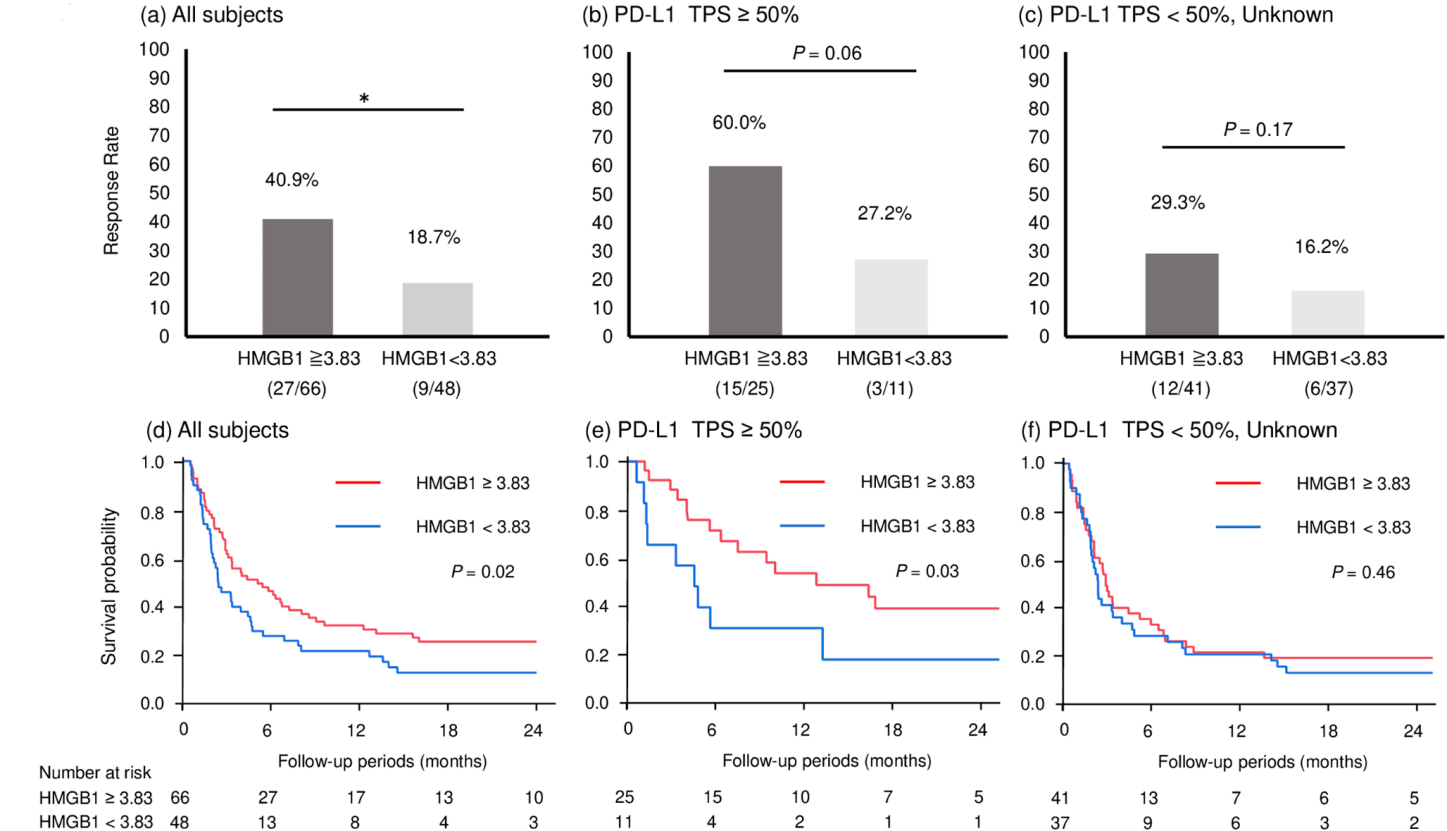
Objective response rate (ORR) and progression free survival (PFS) stratified by serum levels of high mobility group box 1 (HMGB1). In the all subjects, the ORR in the patients with HMGB1 (≥ 3.83 ng/ml) was significantly higher than those without (40.9% vs. 18.7%, *p* = 0.01) (**a**). The same tendency was observed in the patients with NSCLC expressing PD-L1 TPS ≥ 50% (60.0% vs. 22.2%, *p* = 0.06) (**b**), but there was no significant difference in the ORR between the patients with and without HMGB1 (≥ 3.83 ng/ml) in the patients with NSCLC expressing PD-L1 TPS < 50% or unknown (29.3% vs. 16.2%, *p* = 0.17) (**c**). Kaplan–Meier curve analysis revealed that PFS was significantly longer in the patients with HMGB1^high^ than those without (median PFS, 4.3 months vs. 2.3 months, *p* = 0.02) (**d**). The significant difference in PFS was also observed in the patients with NSCLC expressing PD-L1 TPS ≥ 50% (median PFS, 12.4 months vs. 4.4 months, *p* = 0.03) (**e**), but not in the patients with NSCLC expressing PD-L1 TPS < 50% or unknown (median PFS, 2.8 months vs. 2.3 months, *p* = 0.46) (**f**). * *p* < 0.05 using Pearson’s chi-square tests. ORR, objective response rate; HMGB1, high-mobility group box 1; NSCLC, non-small cell lung cancer; PD-L1, anti-programmed cell death ligand-1; TPS, tumor proportion score; PFS, progression free survival

Serum HMGB1 levels were significantly higher in patients with complete response (CR) or partial response (PR) than that in the patients with stable disease (SD) or progressive disease (PD) (5.26 ng/mL [3.62–13.94] vs. 3.87 ng/mL [1.99–8.52], *p* = 0.02; Fig. 2). The optimal cut-off serum HMGB1 level for predicting treatment response as determined by ROC curve analysis (area under the curve = 0.636) was 3.83 ng/mL (Supplementary Fig. 1). The ORR was significantly higher in patients with higher levels of serum HMGB1 (≥ 3.83 ng/mL) than in those with lower levels of serum HMGB1 (40.9% vs. 18.7%, *p* = 0.01; Fig. 3a). In patients with NSCLC expressing PD-L1 TPS ≥ 50%, the ORR tended to be higher in the HMGB1^high^ group than that in the HMGB1^low^ group (60.0% vs. 22.2%, *p* = 0.06; Fig. 3b); however, there was no significant difference in ORR between the HMGB1^high^ and HMGB1^low^ groups in patients with NSCLC expressing PD-L1 TPS < 50% or unknown (29.3% vs. 16.2%, *p* = 0.17; Fig. 3c).

### Predictive accuracy of the combination of HMGB1 and PD-L1 TPS for anti-tumor response

As shown in Table 2a, an HMGB1 cut-off level of 3.83 ng/mL provided a sensitivity of 75.0%, specificity of 50.0%, and positive likelihood ratio (LR) of 1.5 to predict the response to anti-PD-1/PD-L1 antibody monotherapy. High expression of PD-L1 TPS (≥ 50%) provided a sensitivity of 50.0%, specificity of 76.9%, and positive LR of 2.2. Additionally, the combination of HMGB1 and PD-L1 TPS provided higher specificity and positive LR than HMGB1 or PD-L1 alone; the combination provided a sensitivity of 41.7%, specificity of 87.2%, and a positive LR of 3.3. The ORR was the highest (60.0%) in patients with HMGB1 ≥ 3.83 and PD-L1 TPS ≥ 50% in the analyses (Table 2b).

**Table 2:** Association of tumor response with PD-L1 and HMGB1

(a)						
			Sensitivity (%)		Specificity (%)	Likelihood ratio (+)
PD-L1 TPS ≥ 50%			50.0		76.9	2.2
HMGB1 ≥ 3.83 ng/ml			75.0		50.0	1.5
PD-L1 TPS ≥ 50% and HMGB1 ≥ 3.83 ng/ml			41.7		87.2	3.3
**(b)**						
	**PD-L1 TPS**		**HMGB1**		**Combination (PD-L1 TPS and HMGB1)**	
	**≥ 50%**	**< 50%**,** Unknown**	**≥ 3.83 ng/ml**	**< 3.83 ng/ml**	**PD-L1 TPS ≥ 50%** **and** **HMGB1 ≥ 3.83 ng/ml**	**PD-L1 TPS < 50%**,** unknown** **or** **HMGB1 < 3.83 ng/ml**
CR/PR, n(%)	18 (50.0)	18 (23.1)	27 (40.9)	9 (18.8)	15 (60.0)	21 (26.6)
SD/PD, n(%)	18 (50.0)	60 (76.9)	39 (59.1)	39 (81.2)	10 (40.0)	68 (73.4)

PD-L1, programmed death ligand 1; TPS, tumor proportion score; HMGB1, high mobility group box 1; CR, complete response; PR, partial response; SD, stable disease; PD, progressive disease

### Correlation between serum HMGB1 levels and PFS

Our study also revealed that higher serum HMGB1 level was associated with longer PFS. Kaplan-Meier curve analysis revealed that PFS in patients with higher serum HMGB1 levels (≥ 3.83 ng/mL) was significantly longer in comparison with that in patients with lower serum HMGB1 levels (< 3.83 ng/mL) (median PFS, 4.3 months vs. 2.3 months, *p* = 0.02; Fig. 3d).

The significant association between higher level of serum HMGB1 and longer PFS was also observed in patients with NSCLC expressing PD-L1 TPS ≥ 50% (median PFS, 12.4 months vs. 4.4 months, *p* = 0.03; Fig. 3e), but not in patients with NSCLC expressing PD-L1 TPS < 50% or unknown (median PFS, 2.8 months vs. 2.3 months, *p* = 0.46; Fig. 3f).

Univariate cox proportional hazards analysis revealed that higher levels of serum HMGB1 (≥ 3.83 ng/mL) was significantly associated with longer PFS in the entire cohort of patients (HR = 0.62, 95% CI: 0.41–0.94, *p* = 0.025; Table 3) and the patients with NSCLC expressing PD-L1 TPS ≥ 50% (HR = 0.41, 95% CI: 0.18–0.97, *p* = 0.042; Table 3). Additionally, multivariate analysis revealed a significant association only in the patients with NSCLC expressing PD-L1 TPS ≥ 50% (HR = 0.30, 95% CI: 0.12–0.76, *p* = 0.011; Table 3).

**Table 3:** Cox proportional hazard analysis to identify the significant factors prolonging PFS

	All subjects			PD-L1 TPS ≥ 50%			PD-L1 TPS < 50%		
	HR	95%CI	*p*-value	HR	95%CI	*p*-value	HR	95%CI	*p*-value
Univariate analysis									
Age, years	0.99	0.97–1.01	0.284	0.98	0.93–1.03	0.454	1.00	0.98–1.03	0.812
Sex, male	1.16	0.72–1.87	0.533	1.07	0.43–2.62	0.886	1.12	0.64–1.96	0.695
Smoking history, never	1.79	1.07–2.97	0.026*	2.90	1.07–7.90	0.037*	1.40	0.77–2.53	0.267
PS, 0–1	0.65	0.40–1.07	0.091	0.86	0.29–2.52	0.778	0.62	0.35–1.09	0.100
Stage									
III	ref								
IV	1.33	0.68–2.61	0.405	2.43	0.55–10.8	0.241	0.97	0.46–2.08	0.941
Recurrence	1.50	0.72–3.12	0.275	3.59	0.73–17.6	0.114	0.95	0.41–2.17	0.897
Histological type, Sq	0.90	0.57–1.44	0.663	0.72	0.27–1.93	0.508	0.95	0.56–1.61	0.844
PD-L1 TPS, ≥ 50%	0.47	0.29–0.75	0.002*						
Line, 1st	0.42	0.24–0.74	0.003*	0.71	0.31–1.65	0.426	0.25	0.03–1.81	0.170
HMGB1, ≥ 3.83	0.62	0.41–0.94	0.025*	0.41	0.18–0.97	0.042*	0.84	0.52–1.35	0.470
Multivariate analysis									
PD-L1 TPS, ≥ 50%	0.70	0.39–1.26	0.234						
Smoking history, never	1.76	1.04–2.99	0.036*	4.40	1.31–14.7	0.016*	1.62	0.88–2.99	0.123
Line, 1st	0.59	0.30–1.18	0.138	1.07	0.40–2.83	0.891	0.17	0.02–1.31	0.089
PS, 0–1	0.61	0.36–1.01	0.055	0.49	0.15–1.61	0.240	0.56	0.32–0.99	0.049*
HMGB1, ≥ 3.83	0.68	0.44–1.06	0.086	0.30	0.12–0.76	0.011*	0.88	0.54–1.43	0.599

* *p* < 0.05 Cox proportional hazard analysis

PFS, progression free survival; HR, hazard ratio; CI, confidence interval; PS, performance status; Sq, squamous cell carcinoma; PD-L1, programmed death ligand 1; TPS, tumor proportion score; ICI, immune checkpoint inhibitor; HMGB1, high mobility group box 1

## Discussion

This study demonstrated that higher level of HMGB1 (≥ 3.83 ng/mL) is significantly associated with higher ORR and longer PFS in patients with NSCLC who underwent anti-PD-1/PD-L1 antibody monotherapy. Additionally, the association between high levels of HMGB1 and longer PFS was more obvious in the patients with NSCLC expressing PD-L1 TPS ≥ 50%. Therefore, the results of the present study suggest that HMGB1 may be a predictive biomarker of the high efficacy of anti-PD-1/PD-L1 antibody monotherapy and that it may be potentially complementary to PD-L1 TPS to identify patients who would benefit from immunotherapy of anti-PD-1/PD-L1 antibody.

This study demonstrated that serum levels of HMGB1 were significantly higher in the patients with CR/PR compared with that in those with SD/PD, and higher levels of serum HMGB1 (≥ 3.83 ng/mL) were significantly associated with higher ORR and longer PFS. Several studies have demonstrated the association between increased levels of HMGB1 and anti-tumor effects [10, 11]. Rovere-Querini et al. demonstrated that injecting recombinant HMGB1 in a subcutaneous lymphoma mice model inhibited tumor growth [10]. Additionally, HMGB1 is secreted during immunogenic cell death [11], and accelerates DC maturation thereby activating anticancer immunity [9]. HMGB1 also promotes proliferating T cells and suppresses regulatory T cells [12, 13]. Therefore, higher levels of circulatory HMGB1 prior to anti-PD-1/PD-L1 antibody therapy may be associated with acceleration of cancer immunity, thereby improving the efficacy of anti-PD-1/PD-L1 antibody monotherapy.

This study also demonstrated that the combination of HMGB1 and PD-L1 TPS increased the specificity and positive LR to predict the response to anti-PD-1/PD-L1 antibody therapy compared to PD-L1 TPS or HMGB1 alone. Additionally, higher levels of serum HMGB1 (≥ 3.83 ng/mL) were associated with longer PFS particularly in the patients with NSCLC expressing PD-L1 TPS ≥ 50%. Based on these data, a potential mechanism through which HMGB1 promotes anti-PD-1/PD-L1 therapy-mediated anticancer immunity might be explained as follows; in general, cancer immunity cycle, which involves several important steps, is proposed to explain the mechanism of action of ICIs [14]. One of them is cancer antigen presentation. HMGB1 activates cancer antigen presentation by interacting with toll-like receptors expressed on immature DCs [15]. Another step in the cycle is the recognition and killing of cancer cells by T cells, which is suppressed by high expression of PD-L1 on tumors. Therefore, anti-PD-1/PD-L1 antibody is more effective in NSCLC expressing PD-L1 TPS ≥ 50% compared to those expressing PD-L1 TPS < 50% [2]. These data suggest that the high efficacy of anti-PD-1/PD-L1 antibodies in NSCLC expressing PD-L1 TPS ≥ 50% is further accelerated by higher level of HMGB1, because HMGB1 promotes cancer immune cycle by activating cancer antigen presentation, which is a different step in the cancer immunity cycle from those modulated by PD-1/PD-L1 antibodies.

Our study demonstrated the potential of HMGB1 as a biomarker for predicting therapeutic efficacy of anti-PD-1/PD-L1 antibody monotherapy in the patients with NSCLC expressing PD-L1 TPS ≥ 50%. Currently, anti-PD-1/PD-L1 antibodies combined with chemotherapy are widely used as the gold standard for the treatment of advanced NSCLC [16, 17]. However, there is no clear consensus on whether anti-PD-1/PD-L1 antibodies or anti-PD-1/PD-L1 antibodies plus chemotherapy is more suitable for the patients with NSCLC expressing PD-L1 TPS ≥ 50%. This decision should be taken after careful consideration of all factors, because anti-PD-1/PD-L1 antibodies plus chemotherapy is associated with a higher frequency of serious adverse effects than anti-PD-1/PD-L1 antibody monotherapy. HMGB1 may potentially aid in this decision-making. Our future perspective is to investigate whether the combination of HMGB1 and PD-L1 TPS could select patients who should not be treated with co-administration of cytotoxic chemotherapy and who are more suitable for anti-PD-1/PD-L1 antibody monotherapy.

This study had certain limitations. First, this study was conducted in only two institutions, and a replication study is necessary to reevaluate the predictive cut-off level of circulatory HMGB1. Second, this was a retrospective cohort study; thus, most patients were treated with anti-PD-1/PD-L1 antibodies in the second- or later-lines. Further investigations are needed to validate the association between HMGB1 and efficacy in a first-line setting.

## Conclusion

Increased levels of HMGB1 are significantly associated with higher ORR and longer PFS in the patients with NSCLC, especially in those expressing PD-L1 TPS ≥ 50%. Our data also suggest that HMGB1 may be a complementary biomarker to PD-L1 TPS in stratifying patients with NSCLC who may benefit from ICI therapy.

## Electronic supplementary material

Below is the link to the electronic supplementary material.

Supplementary Material 1

## Data Availability

Data are available to interested researchers upon reasonable request to the corresponding author based on ethical approval.

## Abbreviations

AUC: Area under the curve
DC: Dendritic cells
HMGB1: High-mobility group box 1
ILD: Interstitial lung disease
IQR: Interquartile ranges
LR: Likelihood ratio
NSCLC: Non-small cell lung cancer
PD-1: Anti-programmed cell death-1
PD-L1: Anti-programmed cell death ligand-1
TPS: Tumor proportion score
RECIST: Response Evaluation Criteria in Solid Tumors
ROC: Receiver operating characteristic
PFS: Progression free survival
ORR: Objective response rate
CR: Complete response
PR: Partial response
SD: Stable disease
PD: Progressive disease
