# Supplementary material for: HMGB1 assists the predictive value of tumor PD-L1 expression for the efficacy of anti-PD-1/PD-L1 antibody in NSCLC
Source: Cancer Chemother Pharmacol. 2025 Jan 24;95(1):28. doi: 10.1007/s00280-025-04751-2 (PMC11761546; doi:10.1007/s00280-025-04751-2)
Supplement: Supplementary file 1 — Supplementary Material 1 [file 280_2025_4751_MOESM1_ESM.pdf]

Supplementary files

Supplementary Figure S1. Predictive accuracy of high-mobility group box 1 (HMGB1) for treatment response.

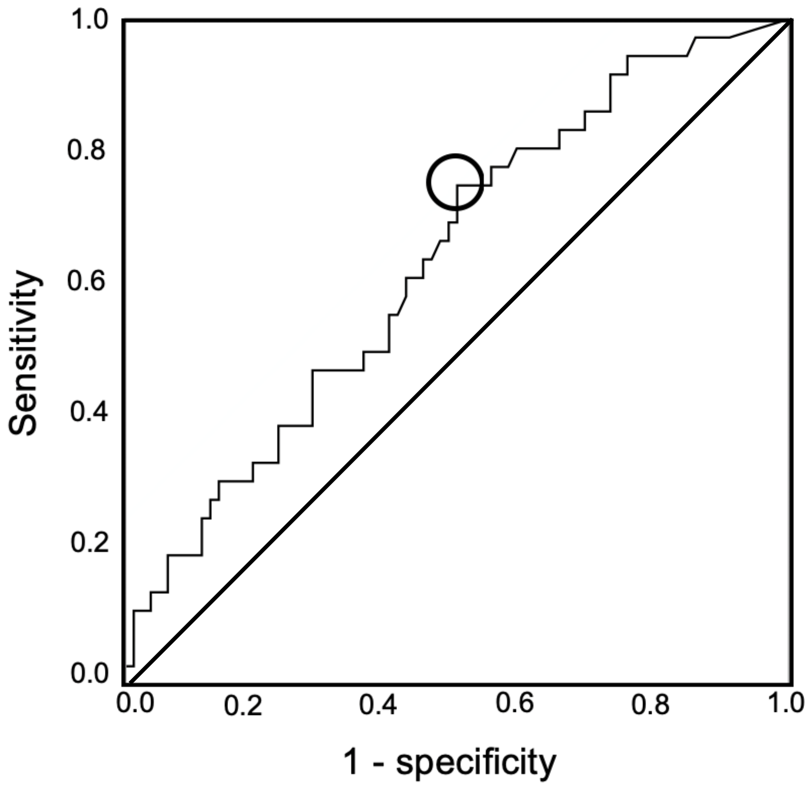

AUC	Cut-off value	Sensitivity	Specificity
0.636	3.83	75	50

Receiver operating characteristic analysis revealed the optimal cut-off level of serum HMGB1 was 3.83 ng/ml for predicting treatment response (AUC 0.636, sensitivity 75%, and specificity 50%).

AUC, area under the curve; HMGB1, high-mobility group box 1.
